# Supplementary material for: Effect-Directed Profiling of Powdered Tea Extracts for Catechins, Theaflavins, Flavonols and Caffeine
Source: Antioxidants (Basel). 2021 Jan 15;10(1):117. doi: 10.3390/antiox10010117 (PMC7830616; doi:10.3390/antiox10010117)
Supplement: Supplementary file 1 [file antioxidants-10-00117-s001.pdf]

Supplementary Material

Effect–Directed Profiling of Powdered Tea Extracts for Catechins, Theaflavins, Flavonols and Caffeine

Gertrud E. Morlock [1,*], Julia Heil [1], Antonio M. Inarejos–Garcia [2], and Jens Maeder [3]

[1] Chair of Food Science, Institute of Nutritional Science, and TransMIT Center for Effect–Directed Analysis, Justus Liebig University Giessen, Heinrich Buff Ring 26–32, 35392 Giessen, Germany

[2] Department of Functional Extracts, ADM Wild Valencia, 46740 Carcaixent, Spain

[3] Department of Science & Technology, ADM Wild Europe, 13597 Berlin, Germany

* Correspondence: Gertrud.Morlock@ernaehrung.uni-giessen.de; Tel.: +49–641–9939140

Dedicated to Joseph Sherma, John D. and Francis H. Larkin Professor of Chemistry Emeritus, Lafayette College, Easton, PA, USA

This pdf contains

Tables S1 to S3

Figures S1 to S6

Table S1 Compilation of the investigated 3 *Camellia sinensis* tea leaf samples and 17 commerical powdered tea extracts on the market (claimed to be aqueous extracts)

**Product category**	**ID**	**Specification**	**Color**
Black tea leaves	**1**	Reference	black
Black tea extract powder	**2**	Not specified	ocre
	**3**	Theaflavins 10%	redish
	**4**	Theaflavins 60%	dark red
	**5**	Polyphenols 20%	light brown–khaki
	**6**	Polyphenols 50%	brown
	**7**	Polyphenols 70%, Theaflavins 40%, Caffeine 1%	red brown
	**8**	Polyphenols 95%	brick red
	**9**	Polyphenols 98%	brick red
White tea extract powder	**10**	Reference	curry yellow
Green tea extract powder	**11**	Polyphenols 15%	ocre
	**12**	Polyphenols 50%	light–brown
	**13**	Polyphenols 90%	dark orange
	**14**	Catechins 15%	ocre
	**15**	EGCg 45%	brick red
	**16**	Catechins 60%	dark orange
	**17**	Catechins 80%	light red–rose
	**18**	Not specified	dark orange
	**19**	Not specified	mustard–yellow
Green tea leaves	**20**	Reference	ocre

Table S2 Compilation of the 32 different mobile phase systems on different RP–18 HPTLC plates (all Merck) investigated for separation of the 11–bioactive–compound mixture (2 µL, 400 ng/band each) up to 6 cm (or 8 cm for No. >16), detected with anisaldehyde sulfuric acid reagent at Vis, or FLD 366 nm to additionally reveal the two flavonols Q and R as light blue fluorescent bands

No.	Solvent composition	Ratio (*V*/*V*/*V*)	Chromatogram	Remark
**RP–18 W (normal phase separation mechanism)**				
1	ethyl acetate – toluene – formic acid	5:5:0.3	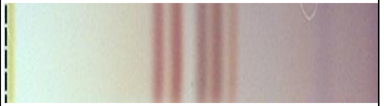	not better than on silica gel
2	**pentyl** acetate – toluene – formic acid	5:5:0.3	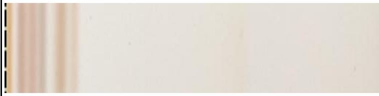	too weak
3	toluene – ethyl acetate – pentyl acetate – formic acid	5:3:2:0.6	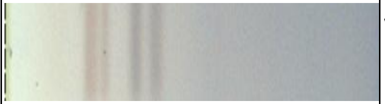	worse
4	ethyl acetate – toluene – formic acid – water	5:1.5:0.6:0.4	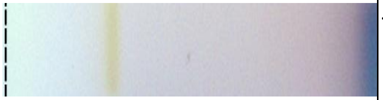	too strong
5	toluene – ethyl acetate – pentyl acetate – **methanol** – formic acid	10:5:5:1.4:0.6	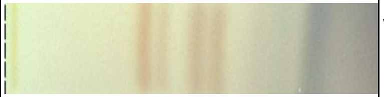	worse
6	toluene – pentyl acetate – **acetonitrile** – formic acid	5:2.5:2.5: 0.3	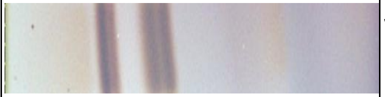	worse

7	***t*–butyl methyl ether** – methanol – water	7:3:1	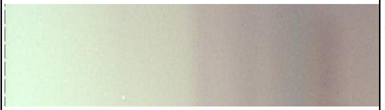	diffuse, took 50 min
	8–10: each with a focusing step up to 1.5 cm with 3 mL methanol to elute R			
8	toluene – pentyl acetate – ethyl acetate –formic acid – water	10:5:5:0.6:0.6	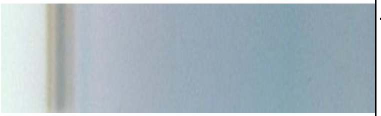	too weak
9	toluene – pentyl acetate – ethyl acetate – formic acid	10:8:2:0.6	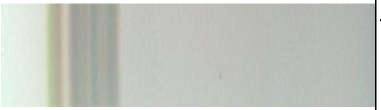	too weak
10	toluene – pentyl acetate – ethyl acetate – formic acid	10:5:5:0.6	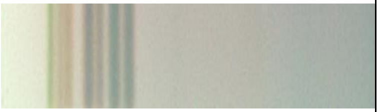	not better than on silica gel
**RP–18 W (reversed phase separation mechanism)**				
11	**acetonitrile** – water – formic acid	5:1:0.2	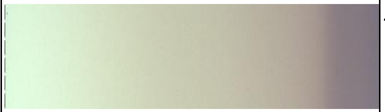	too strong
**12**	**acetonitrile – water – citric acid**	**2.4:8 + 30 mg**	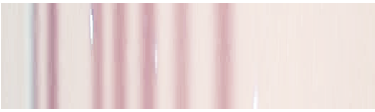	**best on RP–18 W plate**
13	acetonitrile – water – formic acid	1.2:4:0.3	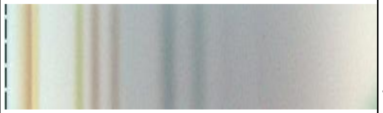	[48]<br>spread over wide range

**RP–18 W F_{254} s (reversed phase separation mechanism)**				
**14**	**acetonitrile – water – citric acid**	**2.4:8 + 30 mg**	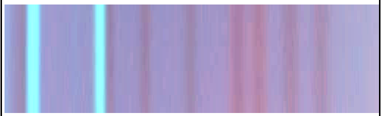	**best on RP–18 W F_{254} s plate**
15	acetonitrile – water – formic acid	1.2:4:0.3	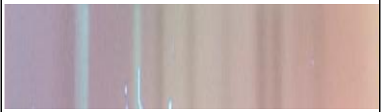	[48]
16	same as 15, but plate prewashed		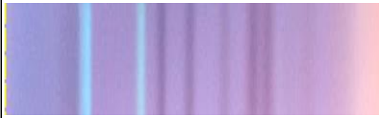	no effect of prewashing
**LiChrospher RP–18 W F_{254} s (normal phase separation mechanism)**				
17	pentyl acetate – ethyl acetate – toluene – formic acid – water	2.5:2.5:5:0.3:0.3	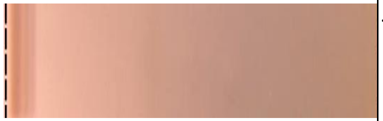	too weak
18	toluene – pentyl acetate – ethyl acetate – formic acid	10:5:8:0.6	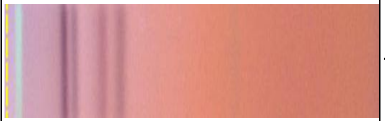	5 compounds<br>too weak

**LiChrospher RP–18 W F_{254} s (reversed phase separation mechanism)**				
19	acetonitrile – water	1.4:4	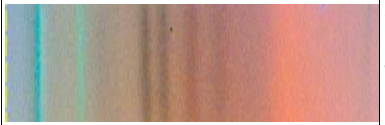	7 compounds<br>diffuse
20	acetonitrile – water – formic acid	1.2:4:0.5	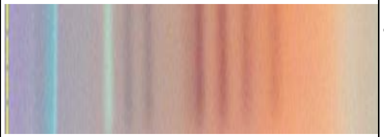	8 compounds
21	acetonitrile – water – formic acid	1.4:4:0.3	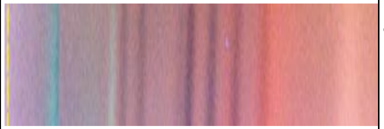	8–9 compounds
22	acetonitrile – water – formic acid	1:4:0.3	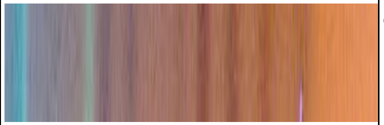	9 compounds
23	acetonitrile – water – formic acid	1.2:4:0.3	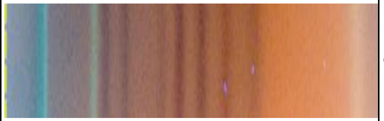	[48]<br>9 compounds
**24**	**acetonitrile – water – citric acid**	**2.4:8 + 30 mg**	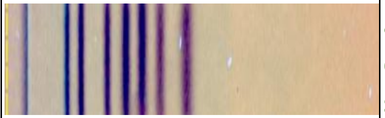	**all 8 flavan–3–ols separated; different color, as reagent sequence was used**

**Machery & Nagel W UV$_{254}$**				
25	acetonitrile – water – formic acid (saturated with + 150 µL toluene)	1.2:4:0.15	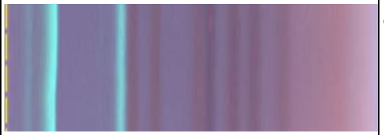	9 compounds
26	acetonitrile – water – formic acid – dimethyl formamide	1.2:4:0.15:0.15	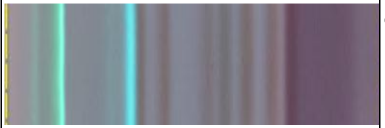	9 compounds<br>background non–homogenously
27	acetonitrile – water – citric acid	2.4:8 + 100 mg	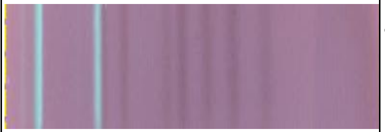	8 compounds
28	acetonitrile – water – citric acid	2.4:8 + 30 mg	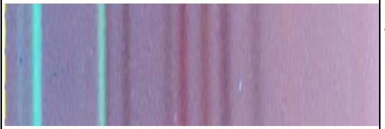	8 compounds
29	acetonitrile – water – formic acid – acetic acid	1.2:4:0.15:0.15	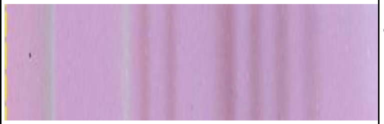	8 compounds
30	acetonitrile – water – formic acid	1.2:4:0.3	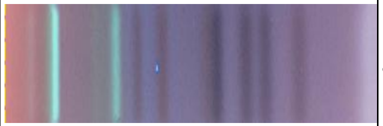	[48]<br>8–9 compounds
**31**	**acetonitrile – water – formic acid**	**1.2:4:0.15**	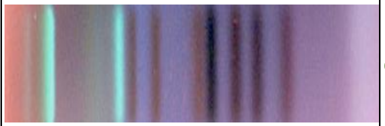	**best on Machery & Nagel plate**<br>**9 compounds**
32	acetonitrile – water – **trifluoroacetic acid**	1.2:4:0.1	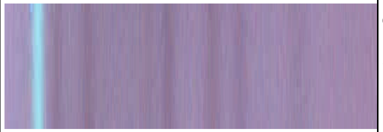	9 compounds<br>Q still on start zone

Table S3 Comparison of different wettable RP–18 HPTLC plate types using the same mobile phase system of acetonitrile – water – formic acid 1.2:4:0.3, *V*/*V*/*V* [48], up to 8 cm, investigated for separation of the 11–bioactive–compound mixture (3 µL, 600 ng/band each) after derivatization with the anisaldehyde sulfuric acid reagent detected at FLD 366 nm to reveal the two flavonols Q and R as light blue fluorescent bands

**Merck**			**Machery & Nagel**		
W	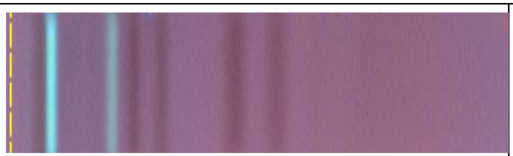	7 compounds	W UV_{254}	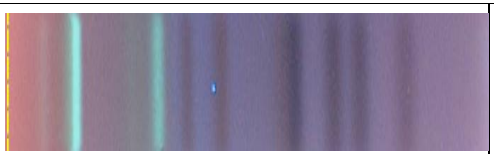	9 compounds
**W F_{254} s**	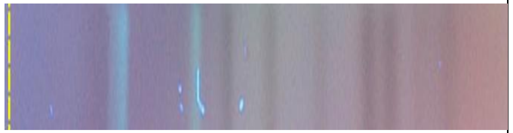	**9–10 compounds**			
Aluminum foil	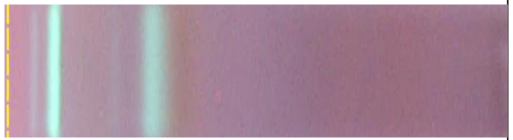	diffuse<br>3 compounds	Aluminum foil	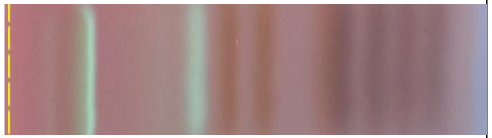	9 compounds
**LiChrospher W F_{254} s**	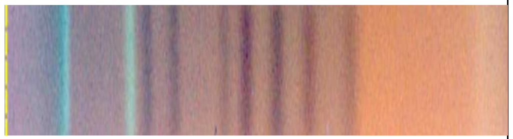	**10 compounds<br>Batch HX42224046**			
	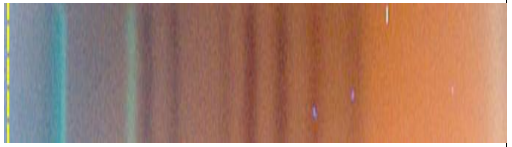	**10 compounds<br>Batch HX602331**			

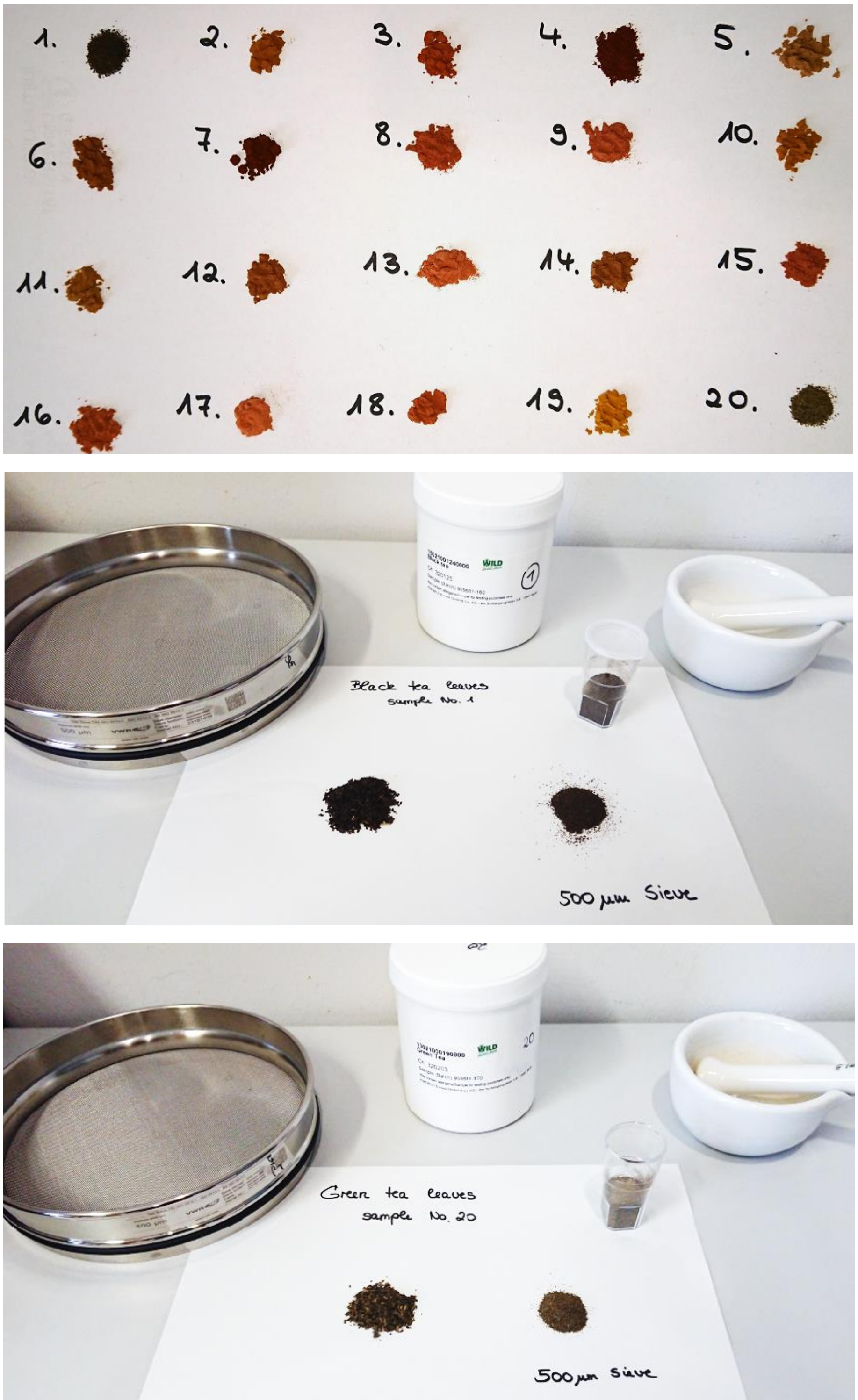

Fig. S1 Images of the investigated 20 *Camellia sinensis* samples for effect–directed profiling: 3 tea leaves (No. 1: black tea, 10: white tea and 20: green tea, all sieved to 500 µm particles, as exemplarily shown) and 17 aqueus tea extract powders.

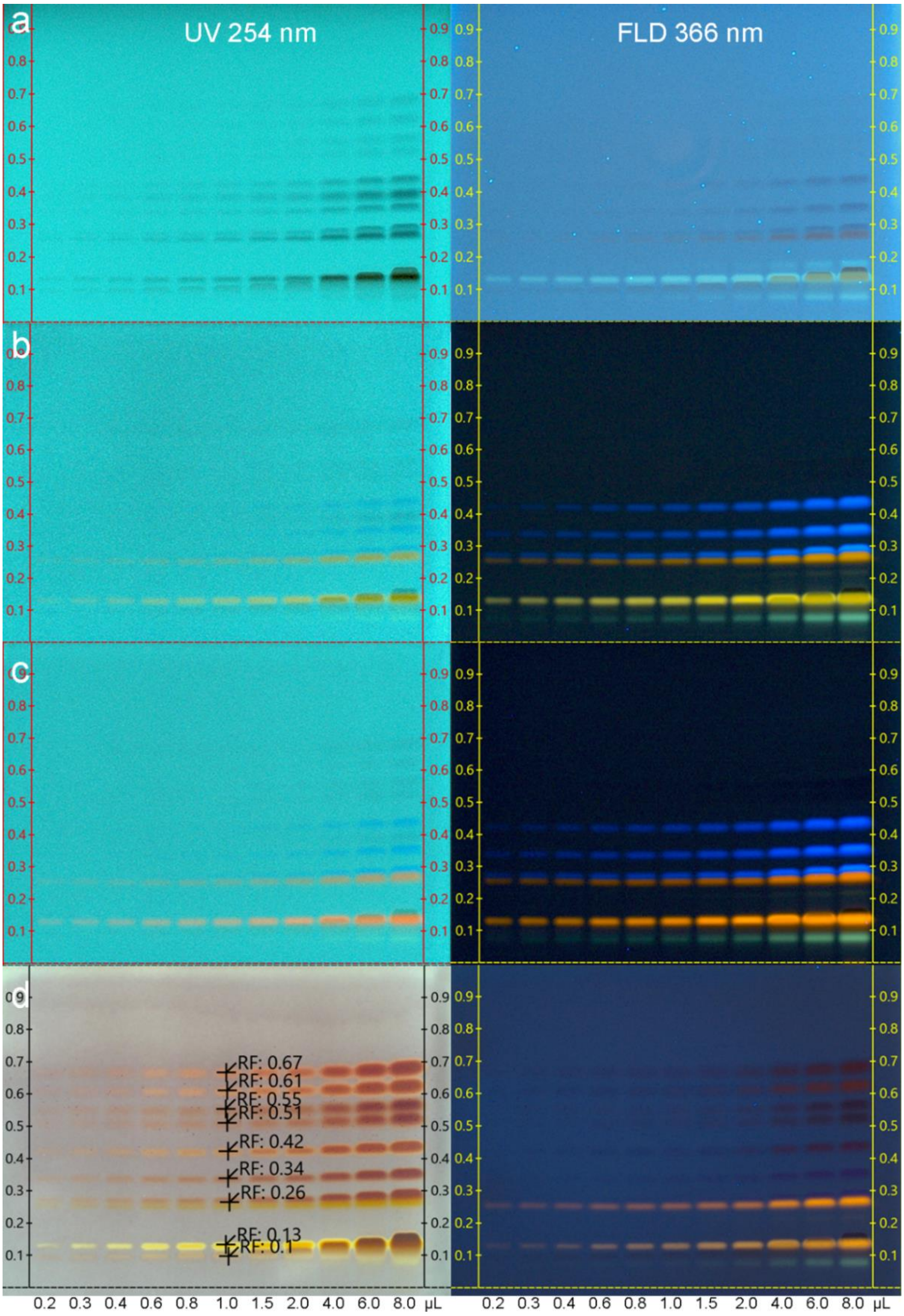

Fig. S2 HPTLC–UV/FLD chromatograms of the amount–dependent separation of the 11–bioactive–compound mixture (0.2–8 µL, 40–1600 ng/band) on the HPTLC plate silica gel 60 RP–18 W F$_{254}$ s (batch HX60386224) using acetonitrile – water – citric acid (1.8 mL + 6 mL + 23 mg) after development (**a**), natural product reagent (**b**), PEG 400 (**c**) and Fast Blue B salt reagents (**d**); the green fluorescent band at hR_F 8 was considered to be an impurity, breakdown product or contaminant.

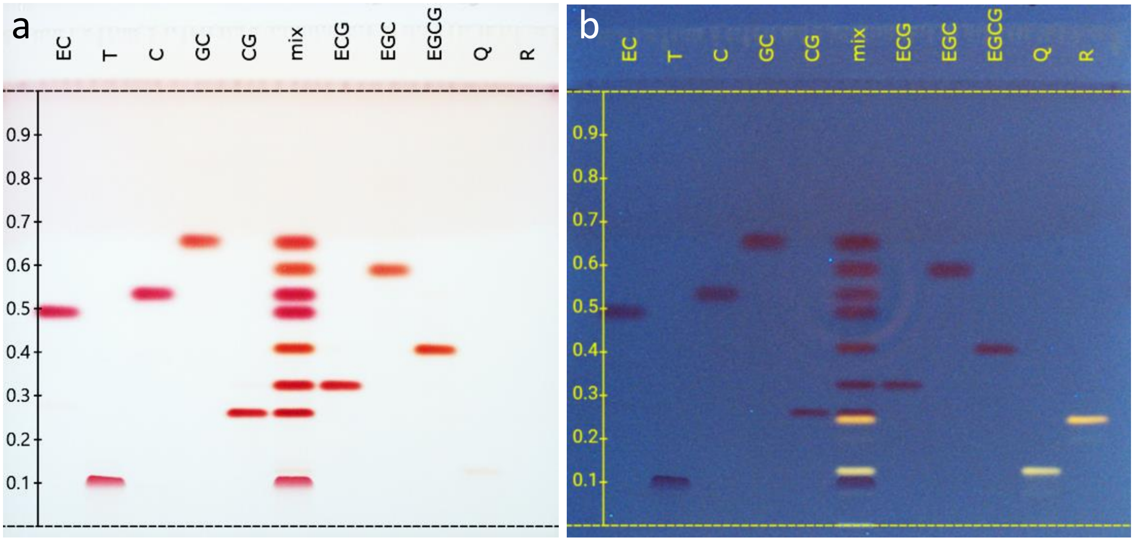

Fig. S3 Assignment of the 10 flavonoids in the 11–bioactive–compound mixture (1 µL, 200 ng/band each): HPTLC–Vis/FLD chromatograms of the separation of the 8 flavan–3–ols and 2 flavonols on the HPTLC plate silica gel 60 RP–18 W F_{254} s (batch HX60386224) using acetonitrile – water – citric acid (1.8 mL + 6 mL + 23 mg), detected after derivatization via a reagent sequence, *i.e.* first applying the Fast Blue B salt reagent (**a**; Vis; only a faint band for Q and none for R), and then, the natural product reagent (**b**; FLD, Q as yellow and R as orange fluorescent band) on the same plate.

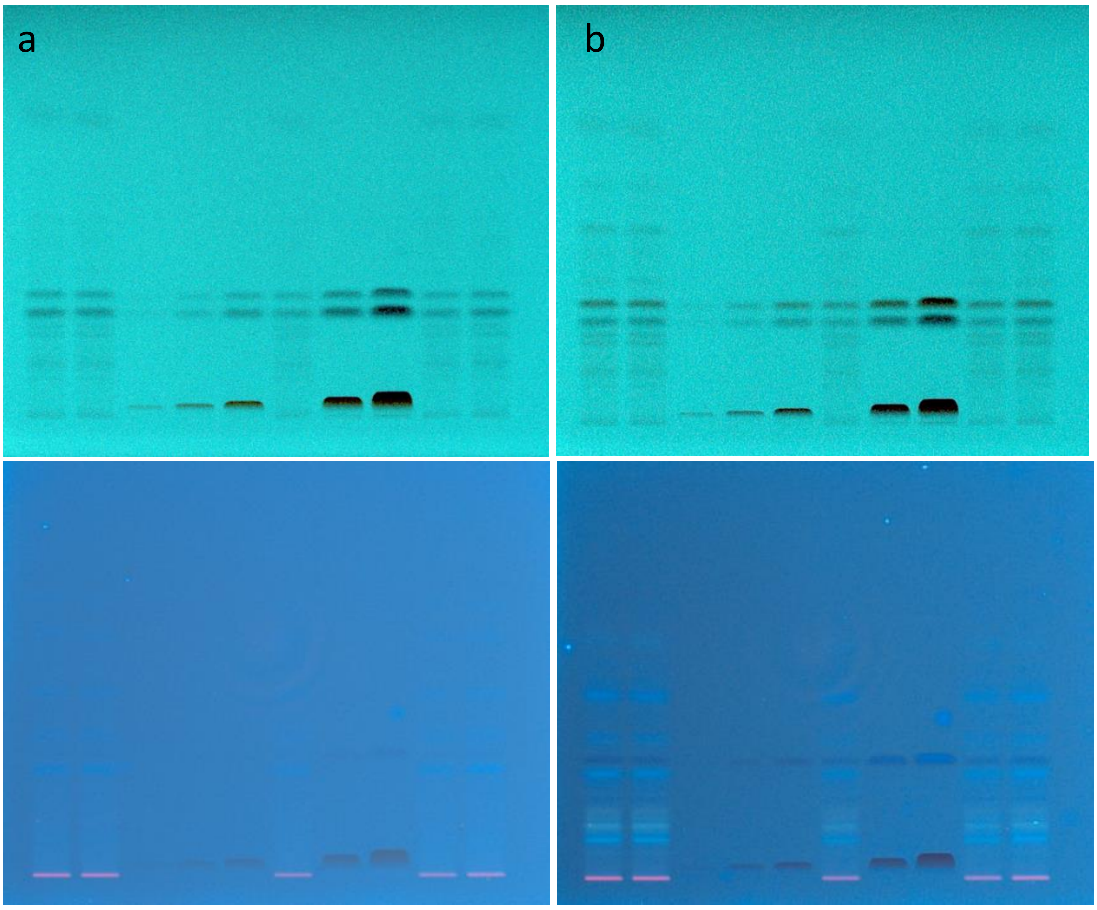

Fig. S4 Response enhancement in the images of the 5–point calibration and quantification of 5 tea samples (TC1–TC5) at UV 254 nm and FLD 366 nm before (**a**) and after the application of the buffer solution (**b**). After development (before application of the assay), the dried chromatogram was neutralized with 2.8 mL sodium hydrogen carbonate buffer (2.5 g/100 mL, pH 8) by piezoelectric spraying (yellow nozzle, level 6) and dried for 4 min.

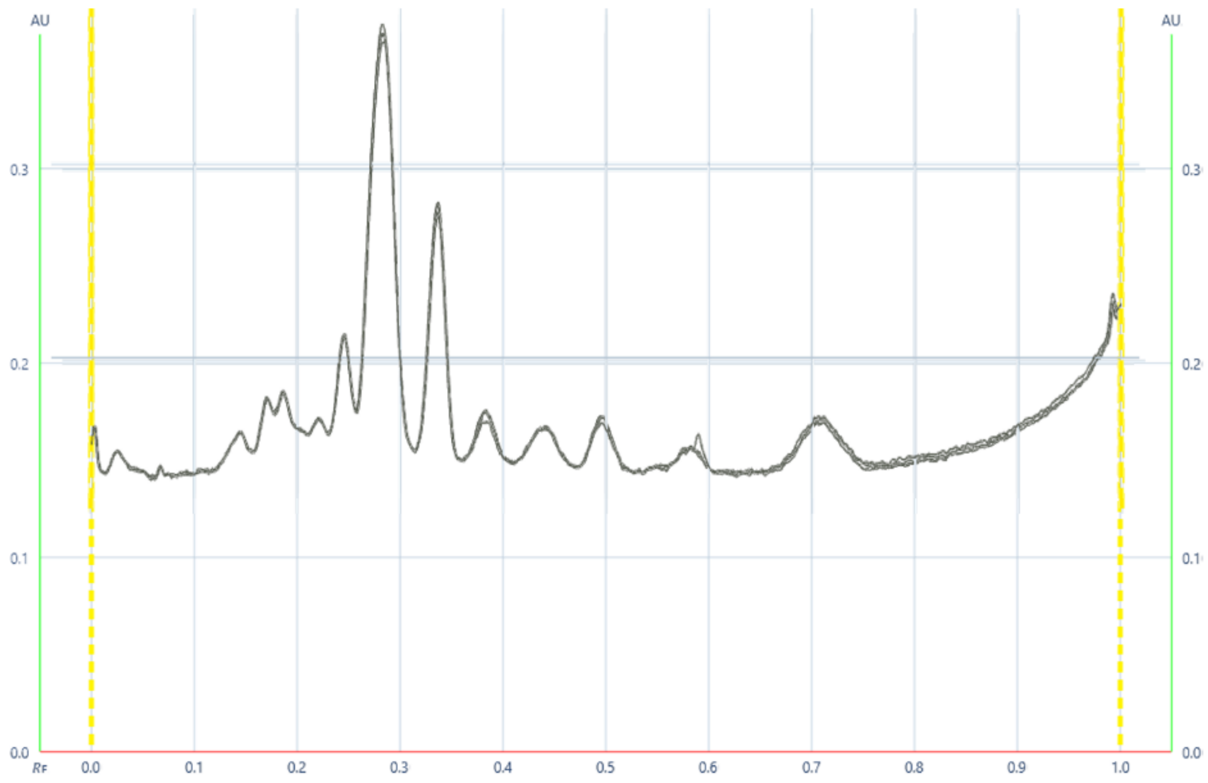

Fig. S5 Overlaid densitometrically measured responses at UV 275 nm, exemplarily shown for the tea sample TC4, obtained directly, after 1 h, 2 h and 3 h on the buffered RP plate proved the stability of the UV signal.

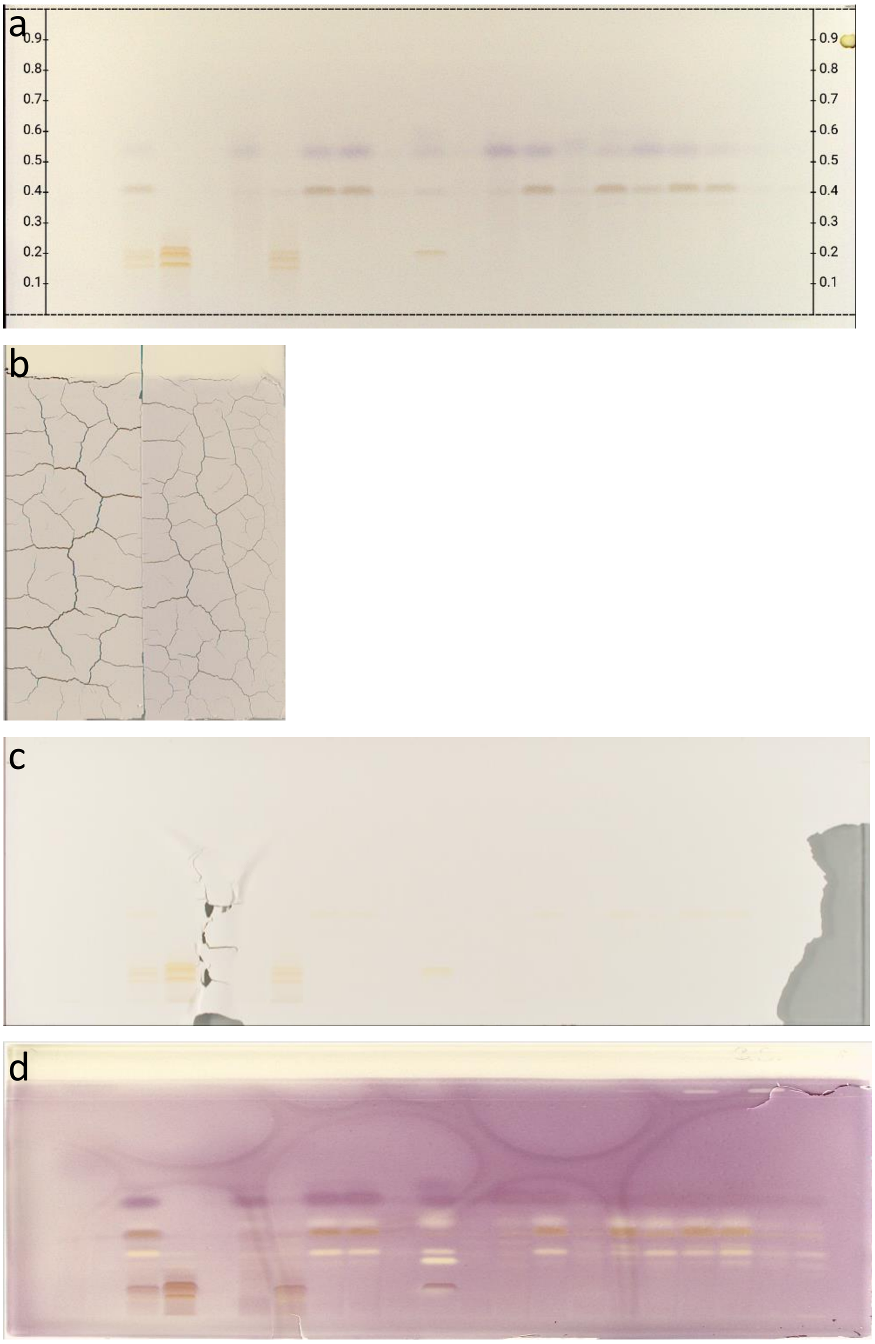

Fig. S6 Observations during the development of the *Bacillus subtilis* bioassay on the LiChrospher® HPTLC plate silica gel 60 RP–18 WF_{254}s: the bioassay was more sensitive to the acidic plate pH of 3.1 after the acidic development and did not lead to the usual background color (a); the use of different plate prewashing protocols (not shown) was not successful as well as a two times plate neutralization with the sodium hydrogen carbonate buffer of pH 8 (b) or a stonger buffer of pH 12 on a twice prewashed plate (c) or on a non–prewashed plate but using overnight incubation (d), all with intermediate plate drying (5 min).
